# The Campaign against Cancer in Missouri

**Published:** 1916-03

**Authors:** 


### The Campaign Against Cancer in Missouri.

The most recent addition to the many agencies, national and local, now engaged in the warfare on cancer is the Department of Preventive Medicine of the University of Missouri. This department has just published in the University bulletin a special article on the early diagnosis and treatment of cancer by Dr. F. A. Martin, instructor in pathology. The purpose of this bulletin is to call attention of its readers in Missouri and elsewhere to the campaign for the education of the laity which is being carried on by the American Society for the Control of Cancer, the American Medical Association and other national and State organizations, and to give a brief general survey of the cancer problem as a phase of preventive medicine.

The knowledge and skill of surgeons in the treatment of cancer has progressed, according to the bulletin, almost to the limits of what is possible, and if the percentage of cures by this, the only method of treatment which offers reliable hope of cure, is to be increased, the patients themselves must co-operate by seeking earlier diagnosis and treatment. On examining the histories of a large number of cases, it has been found that the patients whom the surgeon failed to cure were those who came to him late in the disease, when the cancer had spread to such an extent that to remove all the cancer cells would have required an operation so great that in itself it would be sufficient to cause the death of the patient. On the other hand, it is found of another group of cases which sought treatment soon after the cancer was noticed that 100 per cent were cured. To increase the percentage of cases treated early the University bulletin urges that laymen learn the meaning of cancer and its first warnings in order that they may go to the surgeon in time, when the cancer is still in the early stages and the chance for cure is high.

Among the many facts already known about cancer, perhaps the most important is that the disease nearly always begins in some form of abnormal tissue. This abnormal tissue, which is often easily recognized, may have existed for only a few months or it may have been present from early childhood without causing trouble, only to change into cancer in later life. To these bits of abnormal tissue, or groups of cells, has been given the name of "precancerous lesion." The bulletin says that not all such conditions develop into true cancers, but most of them should be kept under careful observation by a competent medical advisor and removed as soon as there is real danger of malignant diisease. This is the only known method of preventing, as distinguished from curing, cancer, and the Missouri bulletin describes carefully the various forms of precancerous lesions which should be regarded with suspicion. Among these are pigmented moles, cracks on the lip, blisters, scabs and similar persisting abnormal conditions of the skin. Probably only a very small proportion of these conditions become cancer but when moles, for instance, are so located that they are subject to constant irritation and when in later life they change in color and appearance and begin to grow it is time to have them promptly attended to. Moles and warts should never be treated with caustic, but the whole lesion together with its so-called roots should be removed. When a burn on the tongue or lip from smoking does not heal within a few months it is a source of danger. Generally speaking, the removal of precancerous lesions is a trivial operation requiring only local anesthesia.

After true cancer has developed it is still possible to cure a large percentage of cases if the surgeon is given a fair chance while the disease is still local. All cases of cancer are local in the beginning and may remain so far a few weeks to several months. It is during this period that surgical treatment offers the possibility of practically 100 per cent of cures. Unfortunately for the patient, pain is so rare at this stage of the disease and the conditions seem so trivial that in a great number of cases the opportunity to be saved is forfeited by the delay. In cancer of the breast, for instance, the cases cured by the late operation amount to about 30 per cent, but by an early operation at least 80 per cent are saved. If every woman who is not nursing would go to a surgeon within twenty-four hours after she finds a lump in her breast, 90 per cent of the cases of cancer of the breast would be permanently cured.

Cancer of the tongue is perhaps the most malignant, and cures by the late operation are few in number. If a small ulcer appears on the tongue, consult a surgeon at once. When such an ulcer is produced by a ragged tooth, consult a dentist first and then if the ulcer does not heal within a short time after the cause has been removed it is a surgeon's task.

In almost all the common forms cancer is connected with some kind of irritation. Gall stones, for instance, should be removed, since it is established that from 4 to 14 per cent of all cases are followed by cancer.

Cancer of the uterus gives early warning by a discharge of an unusual character at an unusual period and of unusual duration. The removal of the uterus is not a dangerous operation, and if the disease is recognized at an early stage the life of the patient can be saved.

The bulletin issues an emphatic warning against quacks and their bogus testimonials, pointing out that their method of deception lies mainly in the diagnosis. There are so many conditions closely resembling cancer that the average layman cannot distinguish among them, and it is behind such conditions which are not cancer and which would tend to heal without treatment that the "cancer specialists" take their stand and make their false claims.

The Department of Preventive Medicine will supply copies of this cancer bulletin, Medical Series No. 9, upon request to the University of Missouri, Columbia, Mo., as long as the supply lasts.

